# Neural basis of dysphagia in stroke: A systematic review and meta-analysis

**DOI:** 10.3389/fnhum.2023.1077234

**Published:** 2023-01-20

**Authors:** Yin Qin, Yuting Tang, Xiaoying Liu, Shuting Qiu

**Affiliations:** ^1^Department of Rehabilitation Medicine, The 900th Hospital of Joint Logistic Support Force, People’s Liberation Army (PLA), Fuzhou, China; ^2^College of Rehabilitation Medicine, Fujian University of Traditional Chinese Medicine, Fuzhou, China

**Keywords:** stroke, meta, magnetic resonance imaging, dysphagia, activation likelihood estimation

## Abstract

**Objectives:**

Dysphagia is a major cause of stroke infection and death, and identification of structural and functional brain area changes associated with post-stroke dysphagia (PSD) can help in early screening and clinical intervention. Studies on PSD have reported numerous structural lesions and functional abnormalities in brain regions, and a systematic review is lacking. We aimed to integrate several neuroimaging studies to summarize the empirical evidence of neurological changes leading to PSD.

**Methods:**

We conducted a systematic review of studies that used structural neuroimaging and functional neuroimaging approaches to explore structural and functional brain regions associated with swallowing after stroke, with additional evidence using a live activation likelihood estimation (ALE) approach.

**Results:**

A total of 35 studies were included, including 20 studies with structural neuroimaging analysis, 14 studies with functional neuroimaging analysis and one study reporting results for both. The overall results suggest that structural lesions and functional abnormalities in the sensorimotor cortex, insula, cerebellum, cingulate gyrus, thalamus, basal ganglia, and associated white matter connections in individuals with stroke may contribute to dysphagia, and the ALE analysis provides additional evidence for structural lesions in the right lentiform nucleus and right thalamus and functional abnormalities in the left thalamus.

**Conclusion:**

Our findings suggest that PSD is associated with neurological changes in brain regions such as sensorimotor cortex, insula, cerebellum, cingulate gyrus, thalamus, basal ganglia, and associated white matter connections. Adequate understanding of the mechanisms of neural changes in the post-stroke swallowing network may assist in clinical diagnosis and provide ideas for the development of new interventions in clinical practice.

## 1. Introduction

Dysphagia is the inability to deliver food safely and effectively into the stomach due to structural and/or functional impairment of the jaw, lips, tongue, soft palate, pharynx, and esophagus (Wilkinson et al., 2021). Dysphagia is a major cause of death and complications in stroke patients (Vergis et al., 2001; Martino et al., 2005; Takizawa et al., 2016) and has been reported to occur in over 70% of stroke patients (Martino et al., 2005), with at least 75% of stroke patients having moderate to severe early swallowing problems, and 15% of stroke patients still having severe dysphagia after 6 months (Mann et al., 1999). The presence of dysphagia not only interferes with food intake, but also impairs nutrient absorption and leads to malnutrition in patients. Moreover, the patient’s intake during swallowing can deviate from the esophagus into the trachea and affect breathing, leading to aspiration pneumonia (Logemann, 1998). In conclusion, dysphagia not only reduces the quality of life of patients but also leads to high medical costs associated with treatment and care (Takizawa et al., 2016; Patel et al., 2018). Further understanding of the changes in brain neuronal activity that contribute to the emergence of dysphagia can help in the prediction and risk assessment of swallowing characteristics (Daniels et al., 2017), as an adjunct to their clinical diagnosis and treatment, such as providing targets for non-invasive treatments (including repetitive transcranial magnetic stimulation, transcranial direct current stimulation, etc.).

Swallowing is a sensorimotor behavior involving a widely distributed neural network, and is accomplished by the intermodulation of multiple cortical and subcortical sensorimotor brain areas. The sensory-motor areas, insula cortex, parietal temporal lobe, basal ganglia, internal capsule, periventricular white matter (PVWM), and thalamus are thought to be the key cortical and subcortical areas for swallowing after stroke (Cola et al., 2010; Leopold and Daniels, 2010; Wilmskoetter et al., 2020). Alterations in structural and functional networks can help elucidate the relevant neural mechanisms behind dysphagia and provide evidence for stroke-induced functional changes. Structural MRI methods studies have shown that the preservation of structural brain networks (Bonilha et al., 2016) and the location of lesions, correlate with recovery from stroke (Plowman et al., 2012; Hope et al., 2013; Kim et al., 2019). The reason for the association between lesion location and stroke recovery may be the possibility of compensatory neurological recovery, such as the recruitment of new residual brain regions, depending on the damaged brain region (Wilmskoetter et al., 2020). Qiao et al. (2022) performed a qualitative structural neuroimaging analysis of poststroke dysphagia (PSD) and found that the insular cortex, frontal lobe, temporal gyrus, basal ganglia, postcentral gyrus, precentral gyrus, precuneus, and radial corona were the relevant brain areas for dysphagia and the insular cortex probably had the greatest association with PSD and aspiration, but the results obtained lacked quantitative analysis. In related functional MRI methods studies, for example, Mihai et al. (2016) found hyperactivation of the contralateral primary somatosensory cortex but hypoactivation of the bilateral primary motor cortex, supplementary motor cortex, thalamus, and insula in patients with PSD compared to controls. Jiao et al. (2020) found hyperactivation in the vermis cerebelli, right cerebellar hemisphere, left caudate nucleus, left lentiform nucleus, left superior frontal gyrus, and left inferior frontal gyrus, but hypoactivation in the right inferior temporal gyrus, right orbital gyrus, right hippocampus, and right parietal lobe compared to the control group. Liu et al. (2017) conducted a coordinate-based meta-analysis of functional MRI methods studies of PSD, but the volume of included studies was only six, the stability of the results was not high, and more studies need to be included for validation. In summary, it is clear that imaging studies related to PSD yield a large number of brain regions involved in structural lesions and functional abnormalities, and the functional contribution of each brain region has not been adequately tested at this stage.

Therefore, this review will integrate studies using structural and functional imaging methods in stroke dysphagia with a narrative analysis and a quantitative complement using activation likelihood estimation (ALE) methods to integrate the associated structural lesions and functional abnormalities in brain regions that contribute to PSD.

## 2. Materials and methods

Our study was based on the Preferred Reporting Items for Systematic Reviews and Meta-Analyses (PRISMA), which is the minimum set of items reported in an evidence-based meta-analysis (Moher et al., 2009). In addition, this review refers to the practice guidelines for MRI meta-analysis (Müller et al., 2018) (see Supplementary Material 1 for the checklist).

### 2.1. Study selection

All studies were included that met the following criteria: (1) studies that reported PSD using structural and functional MRI methods (PET, SPECT were also included); (2) swallowing screening and/or clinical swallowing assessment and/or instrumental assessment with clear signs of dysphagia or aspiration due to dysphagia; (3) there were no restrictions on language, stroke side or age of participants for inclusion in the study. All relevant studies published up to November 2022 were included.

**FIGURE 1:**
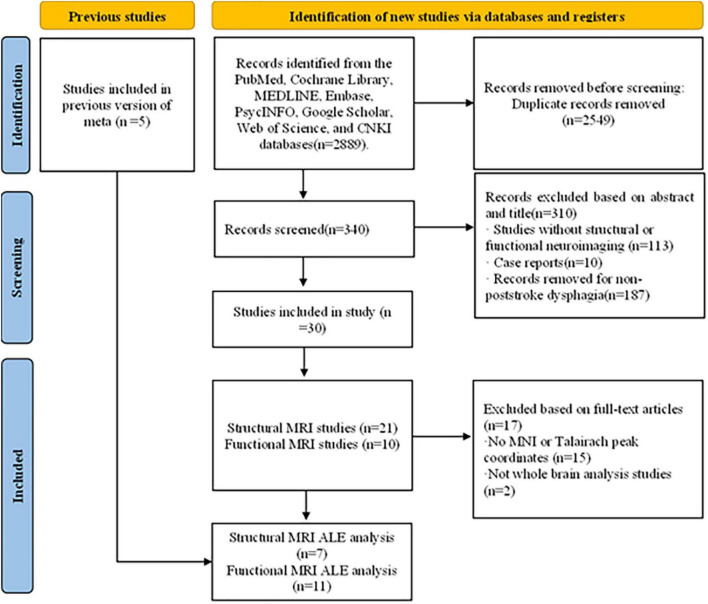
Flow chart of Preferred Reporting Items for Systematic Reviews and Meta-Analyses (PRISMA) study selection process.

A systematic search of the PubMed, Cochrane Library, MEDLINE, Embase, PsycINFO, Google Scholar, Web of Science, and CNKI databases was conducted. The search terms were (“stroke” or “ischemic stroke” or “hemorrhagic stroke”) and (“aspiration” or “abnormal swallowing” or “dysphagia”) and (“magnetic resonance imaging” or “lesion symptom mapping” or “functional magnetic resonance imaging” or “voxel-based image analysis”) and equivalent MeSH terms (see Supplementary Material 2 for the search process). Reference lists of selected studies and related reviews were also retrieved for more comprehensive inclusion. The study selection was carried out independently by Tang and Liu, disagreements were resolved through discussion and negotiation, and a third reviewer, Qiu, was added when they could not be resolved. A total of 340 studies were initially searched and screened, and a total of 30 studies met the inclusion criteria by evaluating the titles and abstracts. Read the full article for further evaluation, which includes a meta-analysis of fMRI of poststroke dysphagia by Liu et al. (2017). After the studies included in their analysis, a new functional ALE analysis was performed on this basis to increase the stability of the results. After including the studies in their analysis, 35 studies were finally included. This included 20 structural neuroimaging and 14 functional neuroimaging studies, and 1 (Mihai et al., 2016) reported both structural and functional neuroimaging (see Figure 1 for a flow chart of the review).

### 2.2. Data extraction

Data extraction for included studies was performed for author information, year of literature publication, number of participants, gender, age, stroke side, imaging method, time from onset to swallowing assessment, and peak coordinates of the Montreal Neurological Institute (MNI) or Talairach reported in the article. If articles reported peak coordinates from the whole brain MNI or Talairach, they were integrated together for the ALE meta-analysis, and studies that did not report coordinates or literature that reported coordinates but region of interest (ROI) studies were included in the narrative analysis. If two or more different tasks are used in the same article, the coordinates of both tasks are included in one dataset. Data extraction was performed independently by Tang and Liu and input into a form designed by the authors. Disagreements were resolved by negotiation through Qiu.

### 2.3. ALE meta-analysis

Activation likelihood estimation is a coordinate based quantitative analysis technique used in neuroimaging. The spatial probability distribution is computed at the center of a given coordinate, and ALE mappings are obtained according to the activation probability of each voxel, distinguishing true convergence of focus from random clustering of focus (i.e., noise) by testing the null hypothesis of random spatial correlation between experiments (Eickhoff et al., 2009, 2012, 2016; Moher et al., 2009; Müller et al., 2018). The ALE meta-analysis method can address the small sample size of a single study, sensitivity to differences between conditions, and limitations such as low reliability, by integrating multiple studies to derive consistency in the location of neuronal changes in the brain (Eickhoff et al., 2009). In this review, structural lesions and functional changes in relevant brain regions were analyzed using the ALE meta-analysis method. The peak coordinates of significant brain regions extracted from all included studies were recorded separately into the specified txt file using Ginger-ALE 3.0.2^1^ (Eickhoff et al., 2009, 2012; Turkeltaub et al., 2012; Müller et al., 2018) software, and the MNI standard space was selected using Lancaster conversion to convert the Talairach coordinates to MIN coordinates. A cluster-level family wise error (FWE) based threshold was selected in reference to Eickhoff et al. (2016), Moher et al. (2009), and the width of the Gaussian probability distribution was determined separately for each experiment based on the peak coordinates of brain regions, based on empirical estimates of inter-subject variability and taking into account the number of subjects in each experiment. The threshold alignment was set to 1,000, the cluster-level FWE value was set to 0.05 and the peak *p*-value threshold was 0.001. Structural MRI ALE analysis was chosen to select larger masks to expand the focal points at the spatial brain boundaries. Functional MRI ALE analysis was chosen to select conservative small mask to limit the experimental effect of adjacent focal points. For the results of the ALE analysis, we examined the contribution of each cluster, and we defined the studies in which the ALE analysis provided coordinates to form clusters as the contributing clusters of studies. The results are shown selected for visualization using Mango 4.1^2^ software, superimposed on the MNI template file at brainmap.org.^3^

#### 2.3.1. Quality assessment and jackknife analysis

We assessed the quality of the included literature using a 10-point checklist produced by Qiu et al. (2021), which included the demographic and clinical characteristics of study participants, image acquisition, and analysis methods. The checklist has been applied in several meta-analyses (Wang et al., 2018; Qiu et al., 2021), and details of the assessment are given in Supplementary Material 3. The assessment was performed independently by Liu and Qiu, and in case of inconsistent scoring a uniform quality score was obtained by discussion. The final quality scores of the study are shown in Table 1. Jackknife analysis tests the replicability of results by manually deleting an experiment and performing ALE analysis on the remaining experiments. Results were considered highly replicable when a brain region was replicated in all or most of the included studies, and in the Jackknife analysis we excluded results that emerged driven by a single or two or three studies. Due to the small number of studies were included, we only report the clusters that were replicated in all jackknife analyses.

**TABLE 1:** Summary of technical and literature quality of studies included in structural activation likelihood estimation (ALE) analysis and functional ALE analysis.

References	Scanners	State	Group comparison	Coordinate	Foci	Quality score
**sMRI**						
Zhang et al., 2021	MRI, VLSM	–	PSD vs. CG	MNI	1	9
Galovic et al., 2016	MRI, VLSM	–	PSD vs. CG	MNI	8	9
Galovic et al., 2017	MRI, VLSM	–	PSD vs. CG	MNI	7	9
Moon et al., 2018	MRI, VLSM	–	PSD vs. CG	Talairach	8	7
Hess et al., 2021	CT, VLSM	–	PSD vs. CG	MNI	11	9
Kim J. et al., 2022	MRI, VLSM	–	PSD vs. CG	MNI	2	8
Hu et al., 2022	MRI, VLSM	–	PSD vs. CG	MNI	1	9
**fMRI**						
Lin et al., 2021	fMRI	Rest	PSD > CG; PSD < CG	MNI	21	8.5
Li et al., 2009	fMRI	Voluntary saliva swallowing task	PSD > CG	Talairach	6	8
Jiao et al., 2020	fMRI	Rest	PSD > CG; PSD < CG	MNI	10	9
* Li et al., 2022	fMRI	Rest	PSD < CG	MNI	5	8.5
Long et al., 2019	fMRI	Rest	PSD > CG; PSD < CG	Talairach	10	8.5
Zhang et al., 2022	fMRI	Voluntary swallowing task	PSD > CG	MNI	10	9
**Liu et al., 2017**						
Osawa et al., 2013	SPECT	–	PSD < CG	Talairach	3	8.5
Momosaki et al., 2012	SPECT	–	PSD < CG	Talairach	5	8
Li et al., 2014a	fMRI	Rest	PSD > CG; PSD < CG	Talairach	12	8
Li et al., 2014b	fMRI	Rest	PSD < CG	MNI	9	7
Malandraki et al., 2011	fMRI	Voluntary swallowing task	PSD > CG	MNI	1	9

*Two datasets are provided. PSD, poststroke dysphagia; CG, control group; sMRI, structural magnetic resonance imaging; fMRI, functional magnetic resonance imaging; VLSM, voxel-based lesion symptom mapping; fMRI, functional magnetic resonance imaging; SPECT, single-photon emission computed tomography; MNI, Montreal Neurological Institute.

## 3. Results

### 3.1. Structural MRI narrative analysis

As can be seen from Table 2 there were 21 studies included in the narrative analysis. The total sample size was 2,447, which included 1,546 patients with dysphagia and 901 controls.

**TABLE 2:** Summary of demographic data for studies included in structural MRI study.

References	Number of subjects (specified per group)	Sample	Age [years, range or *M* ± SD or *M* (IR)]	Sex (Male/Female)	Stroke hemisphere (Left/Right/Bilateral)	Time from onset to swallowing assessment (H/D/M)
^a^ Zhang et al., 2021	PSD	113	67.92 ± 12.22	75/38	43/52/18	NA
	CG	162	63.38 ± 13.19	107/55	84/55/23	
^a^ Galovic et al., 2016	PSD	43	75 ± 15	49/21	22/20/1	<48 h
	CG	76	70 ± 18	43/33	36/35/5	
^a^ Galovic et al., 2017	PSD	25	75 ± 21	28/34	NA	≥7 d
	CG	37				
^a^ Moon et al., 2018	PSD	45	68.02 ± 13.21	57/33	35/50/5	18.9 ± 6.2 d
	CG					
^a^ Hess et al., 2021	PSD	84	73.1 (14.1)	48/36	48/36/0	<48 h
	CG	48	70.5 (14.5)	30/18	25/23/0	
^a^ Kim J. et al., 2022	PSD	50	66.5 ± 10.4	33/17	12/15/23	64.8 ± 38.2 d
	CG		63.2 ± 11.0	26/24	13/17/20	55.2 ± 38.2 d
^a^ Hu et al., 2022	PSD	23	67.2 (11.2)	18/5	7/14/2	<48 h
	CG	103	63.1 (12.1)	76/27	50/48/5	
Jang et al., 2017	PSD	82	73.9 ± 8.01	75/7	11/26/45	35.23 ± 45.89 m
Pyun et al., 2019	PSD	83	70.1 ± 11.6	44/39	36/41/6	21 ± 18 d
Moon et al., 2022	PSD	40	70 (63.5–74)	24/16	10/15/15	35 (21–45) d
Yang et al., 2021	PSD	22	71.9 ± 1.5	20/2	11/11/0	756.1 ± 321.9 d
	CG	18	73.5 ± 1.6	18/0	11/7/0	694.1 ± 197.3 d
Nakamori et al., 2021	PSD	342	70.4 ± 12.6	200/142	173/169/0	<1 w
Galovic et al., 2013	PSD	34	74 (19)	14/20	17/13/4	8 ± 18 h
	CG	60	71.5 (16)	34/26	30/24/6	
Im et al., 2020	PSD	30	67.2 ± 10.2	13/17	0/0/30	14∼26 d
	CG	27	66.3 ± 9.0	11/16	NA	NA
Jang et al., 2020	PSD	20	61.1 ± 12.6	13/7	NA	<6 w
	CG		56.0 ± 10.0	9/11		NA
Domin et al., 2022	PSD	17	56.1 ± 15.6	11/6	8/9/0	NA
	CG		54.5 ± 17.5	9/8	NA	
Kim Y. et al., 2022	PSD	27	68.6 ± 11.2	14/13	14/13/0	<2 w
	CG	24	63.7 ± 11.4	9/15	14/11/0	
Mihai et al., 2016	PSD	18	56.6 ± 15.3	13/5	8/10/0	4∼157 w
	CG		61.94 ± 9.78	4/14	NA	NA
Suntrup-Krueger et al., 2017	PSD	200	73.7 ± 12.2	101/99	102/87/11	<96 h
Suntrup et al., 2015	PSD	200	73.7 ± 12.2	101/99	102/87/11	<96 h
Fandler et al., 2018	PSD	47	70.8 ± 10.8	33/14	NA	0∼9 d
	CG	196	67.1 ± 12.4	123/73	NA	

^a^Study used for activation likelihood estimation (ALE) analysis. PSD, poststroke dysphagia; CG, control group; NA: not available; H, hour; W, week; M, month; M ± SD or M (IR), mean ± standard deviation or median ± interquartile range.

#### 3.1.1. Studies included in the structural MRI ALE analysis

As seen in Tables 1, 2, a total of seven studies were included in the structured ALE meta-analysis. Seven were based on lesion symptom mapping, six were based on MRI imaging of lesion symptom mapping, and one was based on CT imaging of lesion symptom mapping. The total sample size was 904, of which 383 patients with PSD and 521 controls.

In these seven studies, structural brain regions associated with PSD included the left inferior parietal gyrus (Zhang et al., 2021), left frontal lobe (Moon et al., 2018), insular cap (Galovic et al., 2016; Hess et al., 2021), insula (Galovic et al., 2016, 2017; Hess et al., 2021), corona radiata (Galovic et al., 2016, 2017; Hess et al., 2021; Hu et al., 2022; Kim J. et al., 2022), external capsule (Galovic et al., 2016), superior longitudinal fasciculus (Galovic et al., 2016; Hu et al., 2022), internal capsule (Hess et al., 2021), thalamus (Galovic et al., 2017; Hess et al., 2021), primary motor area (Galovic et al., 2017), supplementary motor area (Galovic et al., 2017), and basal ganglia (Galovic et al., 2017; Hess et al., 2021) [including caudate nucleus and lentiform nucleus (Galovic et al., 2016; Kim J. et al., 2022)] are related to other brain regions. By swallowing through site, oral dysphagia was associated with lesions in the insular cap (Galovic et al., 2016) and left frontal lobe (Moon et al., 2018), while pharyngeal dysphagia was associated with lesions in the insular lobe (Galovic et al., 2016), right lentiform nucleus, and right radial corona (Kim J. et al., 2022). In addition, early recovery of swallowing function is associated with damage to the associated white matter of the thalamus and brain (radial corona, etc.), and late recovery is associated with lesions in the functional area of swallowing in the insula (Galovic et al., 2017).

#### 3.1.2. Studies not included in the structural MRI ALE analysis

As seen in Table 2, there were 14 studies that were not included in the structural MRI ALE analysis. The total sample size was 1,543, which included 1,163 patients with PSD and 380 controls. Seven of them reported gray matter brain regions and five were based on whole brain analysis, and two on ROI analysis. Nine studies reported white matter brain regions and two of them (Galovic et al., 2013; Jang et al., 2017) reported both white matter and gray matter regions.

Gray matter brain regions (7 studies) on studies analyzing whole brain gray matter regions, the classification by swallowing through site reported that oral phase dysphagia was associated with lesions in the left inferior frontal lobe (Jang et al., 2017), precentral gyrus (Jang et al., 2017), postcentral gyrus (Pyun et al., 2019), anterior cingulate gyrus (Pyun et al., 2019), caudate nucleus (Pyun et al., 2019), frontal lobe (including medial and inferior frontal gyrus, and frontal part of precentral gyrus), and insula (Pyun et al., 2019). Dysphagia in the pharyngeal phase was mainly associated with lesions in the basal ganglia (Pyun et al., 2019), insula (Pyun et al., 2019), inferior frontal gyrus (Pyun et al., 2019), postcentral gyrus (parietal lobe), and parahippocampal gyrus (Pyun et al., 2019). Classified by the clinical manifestations of dysphagia, impaired residual and swallowing responses were associated with the right parietotemporal region (Suntrup-Krueger et al., 2017) and basal ganglia (Nakamori et al., 2021); impaired cough reflex was associated with the right limbic structures and left sensory area (Suntrup-Krueger et al., 2017); and aspiration was mainly associated with lesions of the putamen (Jang et al., 2017), parietal lobe (Nakamori et al., 2021), right superior temporal gyrus (Suntrup et al., 2015), right superior limbic gyrus (Suntrup et al., 2015), and right temporal plane (Suntrup et al., 2015). In addition, the findings of Suntrup et al. (2015) and Jang et al. (2017) showed that the degree of dysphagia lesions was more correlated with lesions in the right cerebral hemisphere. In terms of two ROI studies, Moon et al. (2022) reported lesion localization analysis of dysphagia after isolated cerebellar stroke and found that the severity of dysphagia was associated with lesions in the left posterior lobe of the cerebellum and that the lesions were more extensive on the left side. Galovic et al. (2013) reported 11 swallowing-related ROIs and found that insular cortex was significantly associated with the risk of post-stroke aspiration.

White matter brain regions (9 studies) several studies have reported that damage to the corticobulbar tract is strongly associated with dysphagia (Fandler et al., 2018; Im et al., 2020; Jang et al., 2020; Kim J. et al., 2022), the internal capsule with aspiration (Galovic et al., 2013; Yang et al., 2021), and the right radial corona with pharyngeal delay (Jang et al., 2017, 2020). Two studies have examined the relationship between somatosensory areas and swallowing, with patients’ corpus callosum and pyramidal tracts connecting primary motor and primary somatosensory cortices integrity and correlated with effective swallowing compliance (Mihai et al., 2016; Domin et al., 2022).

### 3.2. Structural MRI ALE meta-analysis

**FIGURE 2:**
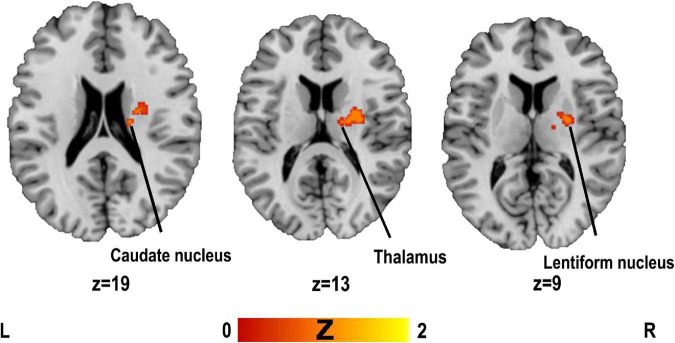
Clusters superimposed on Montreal Neurological Institute (MNI) spatial templates for structural MRI activation likelihood estimation (ALE) meta-analysis.

The results of the ALE analysis showed that two clusters were associated with PSD compared to the control group (see Table 1 for contributing clusters of studies). The brain regions involved are the right lentiform nucleus, the right caudate nucleus and right thalamus (see Figure 2 and Table 3).

**TABLE 3:** Clusters of structural MRI activation likelihood estimation (ALE) meta-analysis.

Cluster	Label	*H*	Volume (mm^3^)	ALE	*Z*	MNI coordinates			*N*	Cs
1	Lentiform nucleus*	R	1,448	0.0142	4.38	20	0	2	4	Galovic et al., 2016, 2017; Hess et al., 2021; Kim J. et al., 2022
				0.0134	4.28	30	−10	14		
				0.0110	3.99	20	−2	−4		
	Thalamus*			0.0111	4.01	20	−12	14		
				0.0107	3.95	16	−12	12		
2	Caudate	R	752	0.0148	4.45	24	−10	24	3	Galovic et al., 2016, 2017; Hess et al., 2021

*Clusters that survived the jackknife analysis. H, hemisphere; R, right; L, left; MNI, Montreal Neurological Institute; N, number of contributions to the cluster; Cs, contributing clusters of studies.

#### 3.2.1. Jackknife analysis

Jackknife analysis showed that the right lentiform nucleus was replicated when deleting either experiment, and the right thalamus and left lentiform nucleus were in a cluster, so we treated them as a whole. The right caudate nucleus did not get replicated when the results of Galovic et al. (2016) or Galovic et al. (2017) were deleted, but this result was not replicated in these two studies from the same laboratory. Thus, the results of the meta-analysis could show a high reproducibility of the right lentiform nucleus and right thalamus.

### 3.3. Functional MRI narrative analysis

As can be seen from Tables 1, 4, a total of 15 studies were included in the narrative analysis of functional imaging MRI. The sample size of the included studies was 715 cases, of which 386 were patients with PSD and 329 were controls.

**TABLE 4:** Summary of demographic data for studies included in functional MRI study.

References	Number of subjects (specified per group)	Sample	Age [years, range or *M* ± SD or *M* (IR)]	Sex (Male/Female)	Stroke hemisphere (Left/Right/Bilateral)	Time from onset to swallowing assessment (H/D/M)
^b^ Lin et al., 2021	PSD	14	40∼80	NA	NA	NA
	CG	14	40∼80	NA	NA	NA
^b^ Li et al., 2009	PSD	10	70.9 (3.4)	5/5	5/5/0	<24 h
	CG	10	70.3 (4.2)	5/5	NA	/
^b^ Jiao et al., 2020	PSD	40	62.9 ± 4.2/63.4 ± 4.6	21/19	19/21/0	3 d
	CG	20	NA	NA	/	/
^b^ Li et al., 2022	PSD	22	60.0 ± 11.38	12/10	NA	<3 m
	CG	30	54.57 ± 10.5	17/13	NA	<3 m
^b^ Long et al., 2019	PSD	20	62.5 ± 3.2	11/9	20/0/0	NA
	CG	20	61.2 ± 2.4	10/10	/	/
^b^ Zhang et al., 2022	PSD	12	63.25 ± 10.30	NA	NA	NA
	CG	21	58.57 ± 10.70	NA	/	/
^b^ Osawa et al., 2013	PSD	27	70.2 (10.3)	13/14	12/4/11	28.9 (11.1) d
	CG	23	71.3 (9.1)	19/4	5/13/5	/
^b^ Momosaki et al., 2012	PSD	10	40.4 (15.8)	8/2	10/0/0	42 (8.6) d
	CG	10	39.4 (9.8)	6/4	/	/
^b^ Li et al., 2014a	PSD	12	50.3 (5.1)	6/6	NA	2∼3 d
	CG	12	51.8 (5.6)	6/6	/	/
^b^ Li et al., 2014b	PSD	10	41.4 (3.7)	NA	5/5/0	3.6 d
	CG	10	43.2 (6.3)	NA	/	/
^b^ Malandraki et al., 2011	PSD	10	38.9 (12.9)	5/5	5/5/0	3∼5 d
	CG	10	35.3 (11.1)	5/5	/	/
Mihai et al., 2016	PSD	18	56.6 ± 15.3	13/5	8/10/0	4∼157 w
	CG	18	61.94 ± 9.78	4/14	NA	NA
Wu et al., 2022	PSD	150	58.07 ± 6.84/ 56.22 ± 7.33	90/60	100/50/0	<48 h
	CG	100	53.56 ± 11.41	57/43	/	/
Yuan et al., 2015	PSD	10	NA	7/3	5/5/0	NA
	CG	10	NA	6/4	/	NA
Dai et al., 2022	PSD	21	59.9 ± 11.1	3/18	15/6/0	<6 m
	CG	21	57.1 ± 7.8	16/5	/	/

^b^Study used for activation likelihood estimation (ALE) analysis. PSD, poststroke dysphagia; CG, control group; NA: not available; H, hour; W, week; M, month; M ± SD or M (IR), mean ± standard deviation or median ± interquartile range.

#### 3.3.1. Studies included in the functional MRI ALE analysis

As can be seen from Tables 1, 4, a total of 11 studies were included in the ALE analysis. The sample size of the included studies was 367 cases, of which 187 were patients with PSD and 180 were controls.

In these 11 studies, functional hyperactivated brain regions associated with PSD included the precentral gyrus (Li et al., 2009), postcentral gyrus (Li et al., 2009), supplementary motor areas (Zhang et al., 2022), cerebellar hemispheres (Malandraki et al., 2011; Li et al., 2014a; Jiao et al., 2020; Lin et al., 2021), cerebellar earth (Jiao et al., 2020), cingulate gyrus (Li et al., 2009; Momosaki et al., 2012; Long et al., 2019; Lin et al., 2021; Zhang et al., 2022), insula (Li et al., 2009, 2014a; Malandraki et al., 2011; Long et al., 2019), thalamus (Zhang et al., 2022), caudate nucleus (Jiao et al., 2020), lentiform nucleus (Jiao et al., 2020; Zhang et al., 2022), superior frontal gyrus (Jiao et al., 2020), inferior frontal gyrus (Jiao et al., 2020; Zhang et al., 2022), visual centers, and primary auditory cortex (Long et al., 2019). Hypoactivation brain regions included precentral gyrus (Long et al., 2019; Lin et al., 2021), postcentral gyrus (Long et al., 2019; Lin et al., 2021), parietal lobe (Jiao et al., 2020; Lin et al., 2021; Li et al., 2022), occipital lobe (Lin et al., 2021), frontal lobe (Lin et al., 2021), insula (Li et al., 2009; Osawa et al., 2013; Long et al., 2019), cuneus (Osawa et al., 2013), cingulate gyrus (Li et al., 2009; Osawa et al., 2013), superior temporal gyrus (Lin et al., 2021; Li et al., 2022), middle temporal gyrus (Li et al., 2022), inferior temporal gyrus (Jiao et al., 2020; Li et al., 2022), orbital gyrus (Jiao et al., 2020), hippocampus (Jiao et al., 2020), and thalamus (Li et al., 2009, 2014a,b,^2022^; Momosaki et al., 2012; Lin et al., 2021), caudate nucleus (Lin et al., 2021), cisternal nucleus (Long et al., 2019), medulla oblongata (Lin et al., 2021), pons (Lin et al., 2021), and posterior cerebellar lobe (Lin et al., 2021). In addition, a study by Long et al. (2019) found that hypoactivation of the precentral gyrus, postcentral gyrus, insula, and putamen was significantly and positively associated with aspiration.

#### 3.3.2. Studies not included in the functional MRI ALE analysis

As seen in Table 4, there were four studies that were not included in the functional MRI ALE analysis. A total of four studies were used for narrative analysis, two of which were whole brain studies, and two of which were ROI studies. One of the studies by Mihai et al. (2016) reported both structural lesions and functional abnormalities. The total sample size was 348, which included 199 patients with dysphagia and 149 controls.

In both studies with whole brain analysis, Wu et al. (2022) found that patients with PSD were hypoactivation in BA4, BA6/8, BA40, and BA13 on the lesion side, and patients had hypoactivation volume of motor function areas and premotor function areas on the lesion side and increased activation volume of motor function areas and premotor function areas on the contralateral side. In contrast, the study by Yuan et al. (2015) found that BA4, BA13, BA40, BA6/8, posterior cingulate cortex (BA23/31), visual association cortex (BA18/19), primary auditory cortex (BA41), parahippocampal gyrus (BA36), somatosensory association cortex (BA7), and left cerebellar regions were overactivated, whereas BA24/32 was hypoactivation in patients compared to controls. In two ROI studies, Mihai et al. (2016) found the hypoactivation in the left and right primary motor cortex, secondary somatosensory cortex, anterior and posterior insula, and affected cerebellum compared to controls, except for hyperactivation in the contralateral primary somatosensory cortex, and asymmetric activation in the ipsilateral cerebellum when the conus tractus lesion was more severe. Dai et al. (2022) studied 20 brain regions associated with swallowing (Dai et al., 2022) examined the functional connectivity of 20 swallowing-related brain regions to the medulla oblongata and found significantly higher functional connectivity of the precuneus, right and left precentral gyrus, and right supplementary motor area to the medulla oblongata.

### 3.4. Functional MRI ALE meta-analysis

**FIGURE 3:**
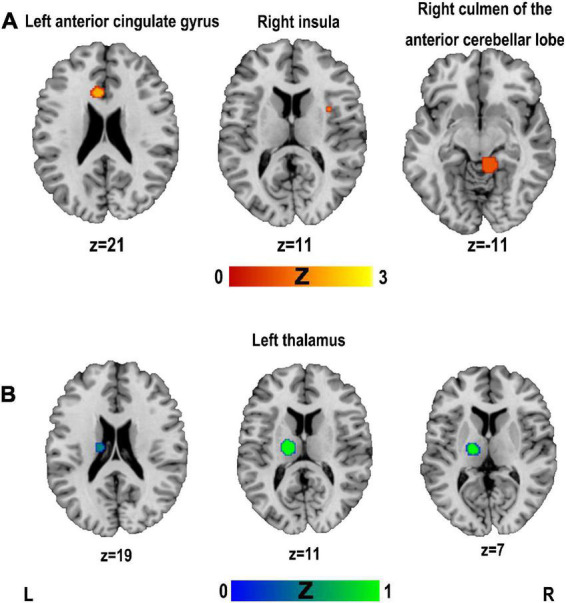
Clusters superimposed on Montreal Neurological Institute (MNI) spatial templates for functional MRI activation likelihood estimation (ALE) meta-analysis. **(A)** The cluster with hyperactivation; **(B)** the cluster with hypoactivation.

Activation likelihood estimation analysis was performed separately for all included studies reporting coordinates of hyperactivation and hypoactivation compared to controls. The results of the ALE analysis showed three clusters of significant hyperactivation compared to controls (see Figure 3A and Table 5). All three clusters had only one peak each and involved the brain regions of the right culmen of the anterior cerebellar lobe, left anterior cingulate gyrus, and right insula. There was one cluster with significant hypoactivation in the left thalamus (see Figure 3B and Table 5).

**TABLE 5:** Clusters of functional MRI activation likelihood estimation (ALE) meta-analyses.

Cluster	Regions	*H*	Volume (mm^3^)	ALE	*Z*	MNI coordinates			*N*	Cs
**Hyperactivation**										
1	Culmen of the anterior cerebellar lobe	R	1,968	0.0015	3.98	10	−40	−10	4	Malandraki et al., 2011; Li et al., 2014a; Jiao et al., 2020; Lin et al., 2021
2	Anterior cingulate gyrus	L	752	0.0012	3.49	−6	22	20	4	Malandraki et al., 2011; Suntrup et al., 2015; Long et al., 2019; Zhang et al., 2022
3	Insula	R	184	0.0011	3.23	36	6	8	4	Li et al., 2009, 2014a; Malandraki et al., 2011; Long et al., 2019
**Hypoactivation**										
1	Thalamus*	L	1,720	0.0015	4.03	−16	−16	12	5	Momosaki et al., 2012; Li et al., 2014a,b, 2022; Lin et al., 2021

*Clusters that survived the jackknife analysis. H, hemisphere; R, right; L, left; MNI, Montreal Neurological Institute; N, number of contributions to the cluster; Cs, contributing clusters of studies.

#### 3.4.1. Jackknife analysis

Jackknife analysis showed that the right culmen of the anterior cerebellar lobe was not replicated when removing Lin et al. (2021) or Jiao et al. (2020). The results of the left anterior cingulate gyrus was not replicated when removing Malandraki et al. (2011) or Zhang et al. (2022). The results of the right insula was not replicated when removing Long et al. (2019). The left thalamus was replicated when removing either study. Therefore, the results of the meta-analysis showed highly replicable in the left thalamus.

## 4. Discussion

We reviewed 21 structural and 15 functional imaging studies on PSD. We used a narrative approach to describe brain regions with structural and functional abnormalities that contribute to PSD, and because of the small number of studies included in the ALE analysis, the results are reported as a supplement, pending further data to draw strong conclusions. The overall results suggest that structural lesions and functional abnormalities in brain regions of the sensorimotor cortex, insula, cerebellum, cingulate gyrus, thalamus, basal ganglia, and associated white matter connections may contribute to dysphagia in individuals with stroke. The ALE analysis provides additional evidence for structural lesions in the right lentiform nucleus and right thalamus, and functional abnormalities in the left thalamus.

According to the anatomical model of swallowing proposed by Daniels and Foundas (1999), swallowing is mediated by a distributed neural network consisting of cortical (including primary motor cortex, primary sensory cortex, premotor and supplementary motor cortical areas, insula, etc.) and subcortical structures (basal ganglia, thalamus, white matter connections, etc.) of the brain, and the various brain areas involved in swallowing have both their own specific functions and are interconnected. We discuss the lentiform nucleus, thalamus, cerebellum and white matter connections, insula, and cingulate gyrus in the following sections.

### 4.1. Subcortical region

Based on the narrative and ALE analyses, it is clear that stroke patients with dysphagia are associated with structural lesions in the right lentiform nucleus, and structural lesions and functional abnormalities in the thalamus. The lentiform nucleus, a component of the basal ganglia, is an important relay nucleus of the inferior cortical conduction tract and plays an important role in maintaining normal tone of the swallowing muscles and in coordinating the swallowing muscle groups (Suntrup et al., 2012). Injury to the lentiform nucleus can lead to damage to the corticomedullary tract, cortico-neostriatum-thalamocortical and neostriatum- substantial nigra loops, resulting in impaired inhibition of the medulla oblongata (Wan et al., 2016). This eliminates the reflex relaxation of the cricopharyngeal sphincter and the sphincter exhibits sustained hyper-reflex contraction which in turn leads to dysphagia. Kim J. et al. (2022) confirmed cricopharyngeal dysfunction and correlated it with the severity of lesions in the lentiform nucleus. In addition, swallowing is a complex sensorimotor process in which the thalamus involves visual, auditory, olfactory, and gustatory sensors and serves as an ingestion center that can be involved in appetitive, endocrine regulation (Toogood et al., 2017). When food enters the mouth, olfactory, and visual responses combine with appetitive responses to activate the brainstem swallowing center (Jie, 2007). During swallowing, the cerebral cortex activates downstream fibers that reach the medulla oblongata swallowing center through the internal capsule and thalamus to produce swallowing and oral mastication movements. Therefore, the cortico-basal ganglia-thalamic circuit (BTC) plays an important role in swallowing, and disruption of this circuit by lesions in any of its components (e.g., structural lesions of the thalamus) may reduce the activation of the thalamus and lead to the development of dysphagia. In addition, related studies have identified thalamic lesions that cause impaired anterior hyoid movement (Wilmskoetter et al., 2019) or delayed pharyngeal swallowing (Wan et al., 2016) leading to dysphagia, and the thalamus may be associated with the prognosis of dysphagia (Galovic et al., 2017).

The cerebellum monitors the execution of swallowing and adapts to planned movements, effectively comparing the expected body movements with the actual behavior and adjusting the movement plan accordingly (Moon et al., 2022). In addition, the cerebellum plays a key role in ensuring accurate, fluid, and coordinated muscle activity (Roostaei et al., 2014) and can effectively coordinate muscle activity associated with swallowing. Several studies on swallowing function in healthy subjects have found various activation patterns in the superior cerebellar hemispheres related to swallowing function (Zald and Pardo, 1999; Suzuki et al., 2003; Malandraki et al., 2009; Grabski et al., 2012). The cerebellum can assist the cerebral cortex in information processing by increasing connections to the cortex and linking to the brainstem to form a cerebellar-cortical pathway (Amore et al., 2021). During voluntary swallowing, the cerebellum acts as a neural response enhancer, modulating or reinforcing swallowing cortical firing, exhibiting functional connectivity with the primary motor cortex, inferior frontal gyrus, basal ganglia, and thalamus (Jayasekeran et al., 2011). These rich functional connections may allow the cerebellum to complement cortical and brainstem control of swallowing after stroke, coordinate cortical and brainstem output, and participate in feedforward and feedback control (Sasegbon et al., 2020).

White matter connections including the corticomedullary tract and the white matter regions through which it passes are involved in motor conduction of swallowing. The corticomedullary tract is the neurotransmission pathway involved in swallowing (Galovic et al., 2017; Jo et al., 2017; Fandler et al., 2018), with fibers from the premotor and primary motor cortex crossing the corona radiata, internal capsule, and pons, and finally projecting to the bilateral cranial nuclei V, VII, IX, X, XI, and XII. Thus, the swallowing muscles are innervated by corticomedullary projections from both hemispheres (Hamdy et al., 1996). Any lesion in this pathway may lead to dysphagia (Jo et al., 2017; Moon et al., 2019; Emos and Rosner, 2022), and post-stroke damage to the corticomedullary tract interrupts the autonomic control of mastication and food mass transport (Cola et al., 2010; Jang et al., 2017), and the more severe the damage, the more difficult is the prognostic recovery of swallowing (Im et al., 2020; Jang et al., 2020).

### 4.2. Insula

Physiological studies have shown that the insula plays a role in regulating sensory and motor aspects of digestive tract function involving the oropharynx, esophagus and possibly other regions of the gastrointestinal tract (Binkofski et al., 1998; Kern et al., 1998; Martin et al., 2001) and autonomous oral motor control (Dronkers, 1996) and that the transition area between the inner surface of the frontal insula and the insula is the primary gustatory cortex (Kobayakawa et al., 1996) and that additional gustatory fields may be located in the insula, temporal lobe and the peduncle of the precentral and postcentral gyrus (Cerf et al., 1998; Faurion et al., 1998; Martin et al., 2001). In addition, the insula receives and projects to brain regions associated with swallowing [including somatosensory-motor cortex, premotor areas, supplementary motor areas, thalamus, anterior cingulate gyrus, and isolated nuclei of the brainstem] (Augustine, 1996; Daniels and Foundas, 1997; Leopold and Daniels, 2010; Wilmskoetter et al., 2020), so the insula is often considered the central hub of the “swallowing network” (Leopold and Daniels, 2010; Babaei et al., 2013; Yuan et al., 2015; Wilmskoetter et al., 2020). Dysphagia due to insula lesions may be physiologically disrupted and associated with disruption of sensory-motor integration (Mosier and Bereznaya, 2001), and there is support for the use of insula lesions in predicting dysphagia (Daniels and Foundas, 1999; Im et al., 2018; Hess et al., 2021). Thus, the insula may be a key region driving neuronal plasticity and effective recovery from dysphagia stroke (Galovic et al., 2017).

### 4.3. Cingulate gyrus

This review also suggests that functional abnormalities of the cingulate gyrus contribute to PSD, which is consistent with the study by Liu et al. (2017). The cingulate gyrus is involved in emotion and reward functions, as well as hunger and taste regulation (Babaei et al., 2010; Long et al., 2019), and is part of the default network, executive control network and the salience network. Therefore, the cingulate gyrus is not only involved in the execution and control of swallowing, but activation of the cingulate gyrus can modulate the negative emotions associated with dysphagia after stroke (Toogood et al., 2017). However, many scholars have also interpreted the involvement of the cingulate gyrus in terms of higher-order motor processing or attention (Hamdy et al., 1999a,b; Martin et al., 2004; Toogood et al., 2005; Malandraki et al., 2009). Toogood et al. (2017) found that brain activation in the anterior cingulate cortex was significantly greater during swallow preparation than during swallow execution, and activation within the bilateral insula and left dorsolateral central cortex was greater during swallow execution than during swallow preparation. Therefore, hyperactivation of the cingulate gyrus after stroke may contribute to an effective recovery of swallowing function (Li et al., 2009; Liu et al., 2017).

### 4.4. Limitations

This review has several limitations. First, the patients included in this review were at different stages of stroke, and the neuropathic mechanisms of dysphagia in acute or subacute vs. chronic stroke may not be the same. Second, the types of stroke included in this review included both ischemic and hemorrhagic strokes, and the location of the lesion was not restricted to a particular brain region, so the neuropathic mechanisms may be different for different stroke types and lesion locations. Furthermore, the number of structural imaging and functional imaging studies included in the ALE analysis was relatively small. When the number of included studies is small, there is a greater likelihood that the data will be driven by a small number of studies and the robustness of the presented results will not be strong enough. We performed a Jackknife sensitivity analysis on the results of the ALE analysis to verify the replicability of the results. We also recognize that the results may change in the future as additional studies on PSD are published. Finally, because ALE meta-analyses can only be performed for studies with whole brain analyses because of the inherent bias in studies of regions of interest, yet the importance of small regions used as ROIs cannot be ignored, future studies need to summarize the main findings of ROI-based studies and acknowledge the possible contribution of these regions. In the future, as larger studies emerge, the inclusion of larger sample sizes for ALE analysis could yield more robust results or allow for subgroup analysis of time period of onset, stroke type, and lesion location; as well as analysis of studies with similar ROIs to provide more insight into the mechanistic contributions of these ROI regions.

## 5. Conclusion

This review provides a comprehensive narrative analysis and ALE complementary analysis of structural imaging and functional imaging studies of PSD. Individuals with stroke have structural lesions and functional abnormalities in brain regions such as sensorimotor cortex, insula, cerebellum, cingulate gyrus, thalamus, basal ganglia, and associated white matter connections that may contribute to dysphagia. The ALE analysis provides additional evidence for structural lesions in the right lentiform nucleus and right thalamus, and functional abnormalities in the left thalamus. These results may contribute to a better understanding of the neuropathophysiological mechanisms in patients with stroke neurological dysphagia and benefit clinical diagnosis and intervention. For example, they provide some ideas for non-invasive neuromodulation therapy (i.e., transcranial magnetic stimulation or transcranial direct current stimulation) targets.

## Author contributions

YQ and YT conceived and designed the study. XL, YT, and SQ were involved in the search and data extraction of the study. YT performed the analysis and wrote the manuscript. YQ critically reviewed the manuscript. All authors approved the manuscript for publication.

## Footnotes

1
http:www.brainmap.org

2
http://rii.uthscsa.edu/mango/

3
http://www.brainmap.org/ale/Colin27_T1_seg_MNI.nii.gz
