# Supplementary material for: Neural basis of dysphagia in stroke: A systematic review and meta-analysis
Source: Front Hum Neurosci. 2023 Jan 20;17:1077234. doi: 10.3389/fnhum.2023.1077234 (PMC9896523; doi:10.3389/fnhum.2023.1077234)
Supplement: Supplementary file 1 [file Presentation_1.pdf]

Checklist for neuroimaging meta-analyses

The research question is specifically defined	YES, and it includes the following contrasts:<br>Poststroke dysphagia patients vs Health Control<br>Poststroke dysphagia patients vs Poststroke patients without dysphagia
The literature search was systematic	YES, a systematic search of the PubMed, Cochrane Library, MEDLINE, Embase, PsycINFO, Google Scholar, Web of Science, and CNKI databases was conducted. It included the following keywords in the following databases: stroke\ ischemic stroke\hemorrhagic stroke AND aspiration\abnormal swallowing\dysphagia AND magnetic resonance imaging\lesion symptom mapping\functional magnetic resonance imaging\voxel-based image analysis. All relevant studies published up to November 2022 were included.
Detailed inclusion and exclusion criteria are included	YES, all studies were included that met the following criteria: (1) studies that reported poststroke dysphagia using structural and functional MRI methods (PET, SPECT were also included); (2) swallowing screening and/or clinical swallowing assessment and/or instrumental assessment with clear signs of dysphagia or aspiration due to dysphagia; (3) compared to controls, there was a whole brain analysis based on Talairach or Montreal Neurological Institute (MNI) spatially significant differences in peak coordinates; and (4) there were no restrictions on language, stroke side or age of participants for inclusion in the study. Region of interest (ROI) or small volume correction (SVC) analyses, as well as studies with other disorders affecting swallowing function (e. g. Parkinson's disease, dementia, etc.) and case studies were excluded.
Sample overlap was taken into account	YES, using the following method: By reading the full article, checking the author's affiliation and the age of publication

All experiments use the same search coverage (state how brain coverage is assessed and how small volume corrections and conjunctions are taken into account)	YES, the search coverage is the following: The literature clearly defines the detailed scanner parameters provided in the whole brain analysis or reading literature, including slice thickness, slice number, gap and visual field (optional FOV: matrix and voxel size). In other cases, experiments that lack only one or two slices can also be considered.
Studies are converted to a common reference space	YES, using the following conversion(s): The Montreal Neurological Institute (MNI) standard space was selected, using Lancaster conversion will convert the Talairach coordinates to MIN coordinates.
Data extraction have been conducted by two investigators (ideal case) or double checked by the same investigator (state how double-checking was performed)	YES, the following authors:<br>Tang AND Qin checked inclusion criteria<br>Tang and Liu extracted coordinates<br>Liu and Qiu extracted other info: Quality assessment.

The paper includes a table with at least the references, basic study description (e.g. for fMRI tasks, stimuli), contrasts and basic sample descriptions (e.g. size, mean age and gender distribution, specific characteristics) of the included studies, source of information (e.g. contact with authors), reference space	YES, and also the following data: Quality assessment scores.
The study protocol was previously registered and all analyses planned beforehand, including the methods and parameters used for inference, correction for multiple testing, etc	1) No registration (COVID-19 is preferred for registration now), but there is a plan in advance.<br>2)Any non-planned analyses are clearly stated as post-hoc or non-prespecified in the paper.<br>3)The meta-analysis used the default methods and parameters of the software.
The meta-analysis includes diagnostics	YES, the following: We manually perform Jackknife analysis of the analysis results.

Müller VI, Cieslik EC, Laird AR, Fox PT, Radua J, Mataix-Cols D, Tench CR, Yarkoni T, Nichols TE, Turkeltaub PE, Wager TD, Eickhoff SB. Ten simple rules for neuroimaging meta-analysis. Neurosci Biobehav Rev. 2018 Jan;84:151-161. doi: 10.1016/j.neubiorev.2017.11.012. Epub 2017 Nov 24. PMID: 29180258; PMCID: PMC5918306.
