# Supplementary material for: Neural basis of dysphagia in stroke: A systematic review and meta-analysis
Source: Front Hum Neurosci. 2023 Jan 20;17:1077234. doi: 10.3389/fnhum.2023.1077234 (PMC9896523; doi:10.3389/fnhum.2023.1077234)
Supplement: Supplementary file 2 [file Presentation_2.pdf]

Contents of the supplementary material

1. Search process

A systematic search of the PubMed, Cochrane Library, MEDLINE, Embase, PsycINFO, Google Scholar, Web of Science, and CNKI databases was conducted. The search terms were ("stroke" OR " ischemic stroke " OR " hemorrhagic stroke ") AND ("aspiration" OR "abnormal swallowing" OR "dysphagia") AND ("magnetic resonance imaging" OR "lesion symptom mapping" OR " functional magnetic resonance imaging " OR "voxel-based image analysis") and equivalent MeSH terms.

Search expressions:

1) ((dysphagia [Title/Abstract]) AND (stroke [Title/Abstract])) AND (network [Title/Abstract] OR fMRI [Title/Abstract] OR DTI [Title/Abstract])

2) (dysphagia OR odynophagia OR (globus sensation) or (globus pharyng-Eus)) AND (network [Title/Abstract] OR fMRI [Title/Abstract] OR DTI [Title/Abstract])

3) dysphagia AND 'functional magnetic resonance imaging':ti,ab,kw

4) dysphagia AND 'computed tomography':ti,ab,kw

5)（Dysphagia OR （Swallowing Disorders) ）AND (network OR fMRI OR DTI)

6)（Swallowing Disorders) AND (network OR fMRI OR DTI)

7) (dysphagia OR (Swallowing Disorders)) AND (magnetic resonance imaging).
