# Supplementary material for: Neural basis of dysphagia in stroke: A systematic review and meta-analysis
Source: Front Hum Neurosci. 2023 Jan 20;17:1077234. doi: 10.3389/fnhum.2023.1077234 (PMC9896523; doi:10.3389/fnhum.2023.1077234)
Supplement: Supplementary file 3 [file Presentation_3.pdf]

Quality Assessment Checklist (When criteria were partially met, 0.5 points were assigned)

**Category 1: Subjects**	Score (0/0.5/1)
1. Patients evaluated prospectively; specific diagnostic criteria applied; demographic data reported.	
2. Comparison subjects evaluated prospectively; psychiatric and medical illnesses excluded; demographic data reported.	
3. Important confounds (e.g. age, gender, IQ) controlled either by stratification or statistically.	
4. Sample size per group > 10.	
**Category 2: Methods for image acquisition and analysis**	
5. Whole brain analysis automated with no a priori regional selection.	
6. Coordinates reported in a standard space.	
7. Imaging technique clearly enough described to be reproduced.	
8. Measurements clearly enough described to be reproduced.	
**Category 3: Results and conclusions**	
9. Statistical parameters provided for significant and important non-significant differences.	
10. Conclusions consistent with results; limitations discussed.	
	TOTAL /10
